# 3D Bioprinting of Gelatin–Xanthan Gum Composite Hydrogels for Growth of Human Skin Cells

**DOI:** 10.3390/ijms23010539

**Published:** 2022-01-04

**Authors:** Beatrice Piola, Maurizio Sabbatini, Sarah Gino, Marco Invernizzi, Filippo Renò

**Affiliations:** 1 Innovative Research Laboratory for Wound Healing, Health Sciences Department, Medical School, Università del Piemonte Orientale, Via Solaroli 17, 28100 Novara, Italy; 20021634@studenti.uniupo.it (B.P.); sarah.gino@uniupo.it (S.G.); 2 Department of Sciences and Technological Innovation, Università del Piemonte Orientale, Via T. Michel 11, 15121 Alessandria, Italy; maurizio.sabbatini@uniupo.it; 3 Health Science Department, Physical Medicine and Rehabilitation Division, Università del Piemonte Orientale, Via Solaroli 17, 28100 Novara, Italy; marco.invernizzi@med.uniupo.it; 4 Department of Integrated Research and Innovation, Translational Medicine Unit (DAIRI), Hospital “S.S. Antonio e Biagio e Cesare Arrigo”, 15121 Alessandria, Italy

**Keywords:** bioprinting, hydrogel, xanthan gum, gelatin, biocompatibility

## Abstract

In recent years, bioprinting has attracted much attention as a potential tool for generating complex 3D biological constructs capable of mimicking the native tissue microenvironment and promoting physiologically relevant cell–cell and cell–matrix interactions. The aim of the present study was to develop a crosslinked 3D printable hydrogel based on biocompatible natural polymers, gelatin and xanthan gum at different percentages to be used both as a scaffold for cell growth and as a wound dressing. The CellInk Inkredible 3D printer was used for the 3D printing of hydrogels, and a glutaraldehyde solution was tested for the crosslinking process. We were able to obtain two kinds of printable hydrogels with different porosity, swelling and degradation time. Subsequently, the printed hydrogels were characterized from the point of view of biocompatibility. Our results showed that gelatin/xanthan-gum bioprinted hydrogels were biocompatible materials, as they allowed both human keratinocyte and fibroblast in vitro growth for 14 days. These two bioprintable hydrogels could be also used as a helpful dressing material.

## 1. Introduction

Hydrogels are crosslinked, insoluble and hydrophilic polymers that are capable of containing a large amount of water thanks to their porosity and three-dimensional network structure [1]. The hydrophilic capacity of hydrogels is due to the presence of hydrophilic groups along the polymer chain, while crosslinks can be built by electrostatic dipole–dipole interactions and covalent bonds [2]. In recent years, hydrogels have aroused a significant interest in biomedical and clinical research due to their capacity to create a favorable microenvironment for cell growth and/or differentiation [3]. Certainly, they have several application domains, such as drug delivery [4,5], tissue engineering [2,6,7], regenerative medicine [8,9] and wound dressings [10,11,12]. In particular, hybrid composite hydrogels (made by gelatin, xanthan gum, glutaraldehyde and HPLC-grade water) have been shown to exhibit good wound-healing ability [13].

Gelatin (Gel), which is produced by partial hydrolysis of collagen, displays good biocompatibility and biodegradability properties and is commonly used in medical areas, due to its great biological advantages and low cost [14]. Several studies have highlighted the beneficial effects of Gel for migration, adhesion and growth of cells in tissue regeneration processes [14,15,16]. Moreover, its biodegradability is the result of the presence of matrix metalloproteinase (MMP) cleavage sites, and it is an important characteristic for the development of in vivo implanted hydrogels, since scaffold degradation allows for the deposition and production of a new extracellular matrix [17].

Xanthan gum (Xnt) is a microbial-derived high-molecular-weight heteropolysaccharide that is employed in medical field and tissue engineering for its biocompatibility and gelling characteristics [18], as well as being available at a low cost and easy to process [19]. For these important properties, it has been widely used in the pharmaceutic field as a thickener, suspender and emulsifier [20] and also in the manufacture of biodegradable hydrogels for skin scaffold [18,21].

An important limit of gelatin and xanthan-gum hydrogels is their mechanical weakness and dissolution behavior in solvent [17]. It is known that, after immersion in water, they present a severe swelling rate, due to the absorption of the solvent. This is a disadvantage for its employment as wound scaffold, because severe swelling will make the dressings unable to maintain the structure of the dressings and fit the wound [14]. To solve this problem, hydrogels are stabilized by crosslinking to increase their material strength and hydrolysis resistance, and to keep their shape, avoiding swelling phenomena [22]. Through crosslinking, it is possible to stabilize the chemistry of the polymer, extending the chains, with consequent modifications of the network structure [23].

Crosslinking agents can be inserted into hydrogels through different methodologies, such as physical methods and the use of chemicals or enzymes [24]. Among the chemical methods, glutaraldehyde (GTA) [25,26] is one of the most used crosslinking agent in the biomedical sector, despite its cytotoxicity at certain concentrations [25]. Crosslinking of collagen derivatives with GTA includes the production of permanent junctions by reactions between free amine groups of lysine or hydroxylysine amino acid residues in polypeptide chains and aldehyde groups of glutaraldehyde [23,27]. Moreover, GTA is easily available at a low cost, and in aqueous solution, it allows crosslinking to be achieved in a relatively short time [27].

Three-dimensional bioprinting is an early stage technology with important consequences and applications in the biomedical and tissue-engineering fields, as it allows the creation of 3D tissue constructs through programmed models and the distribution of the so-called bio-ink by means of the movement of a motorized stage [28,29]. Bio-inks have different compositions (biomaterials, biomolecules and cell) depending on their use; in fact, properties such as printability, biocompatibility and physical strength could affect the final printed construct [30]. Then, the printed constructs could be crosslinked after bioprinting to stabilize its final shape and structure. This technique allows for the standardization of the bioprinting process, with high reproducibility and precise control, potentially enabling high-throughput production [29]. However, current 3D bio-printing methods still have technical limitations to overcome, such as high-resolution cell deposition and controlled cell distribution [28].

Considering this, the aim of present study was to develop a low-cost crosslinked 3D printable hydrogel based on biocompatible natural polymers (gelatin and xanthan gum) and characterize it as a cell-growth scaffold to use for both in vitro growth of tissues and wound dressing.

## 2. Results

### 2.1. Bioprinting and Crosslinking

As reported in Table 1, different concentrations of Gel/Xnt were tested to achieve maximum printability. In the framework of extrusion bioprinting, hydrogel printability generally refers to the extrudability, filament formation and shape fidelity. By modulating the process and 3D printer parameters, it was possible to obtain predictable structures, as were designed in the 3D printing software. The Slic3r program was used to modify the code for a tissue model in order to optimize fill pattern, speed, density and extrusion [31]. A 25-gauge (0.25 mm aperture) printer tip with pneumatic extrusion pressures below 21 kPa was used. Gelatin (2.5 w/v% and 3 w/v%), along with xanthan gum (1.2 w/v%), resulted in being printable and showed the best resolution, intended as shape fidelity, using a pressure range of 10 to 20 kPa.

After the printing process, three different concentrations (0.3, 0.5 and 1 v/v%) of glutaraldehyde and two different immersion times (1 and 3 h) were used for the hydrogel crosslinking test. The higher concentrations of glutaraldehyde, with a longer immersion time, led to greater resistance and permanence of the print shape, which could adversely affect the biodegradability of the printed hydrogels. Therefore, in order to reduce the excessive tribological qualities (durability) [25,32], and also to minimize its possible toxicity [24,25,32], the low concentration of glutaraldehyde was assessed. The 3Gel4 and 2.5Gel3 hydrogels crosslinked with 0.3 v/v% GTA for 3 h resulted in the best stability in PBS and DMEM at 37 °C (Table 2), maintaining their shape for over 48 h, not disintegrating even after a week of immersion.

### 2.2. Characterization of 3D-Printed Hydrogel

#### 2.2.1. Morphology

**Figure 1:**
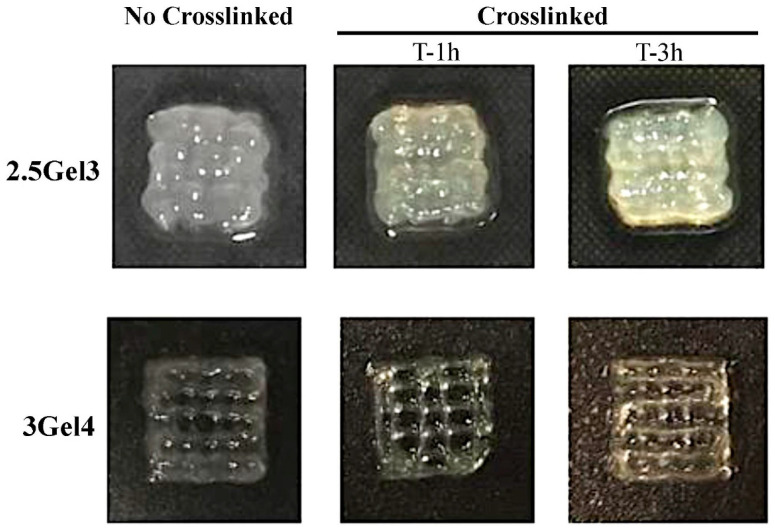
Morphological analysis of the 2.5Gel3 and 3Gel4 hydrogels after printing (not crosslinked) and after submersion for 1 and 3 h in the 0.3 v/v% glutaraldehyde (crosslinked). Hydrogels were printed in a 1 cm^2^ square shape.

Figure 1 shows the images obtained by the morphological analysis of 1 cm^2^ square shapes obtained by extrusion bioprinting. A comparison of the different hybrid composites hydrogels showed that both 3Gel4 crosslinked and non-crosslinked have a defined shape compared to 2.5Gel3, which appears to be less viscous. These results showed that xanthan gum is a good stabilizing agent, because the hydrogel with 3 w/v% of Gel without crosslinker is able to maintain its print shape, which even improves after the immersion in GTA.

#### 2.2.2. Moisture

**Figure 2:**
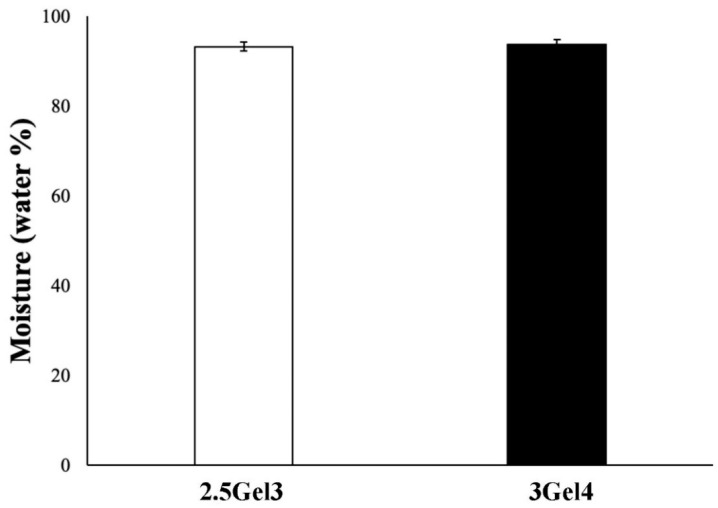
Moisture percentage of 2.5Gel3 and 3Gel4 hybrid composite hydrogels after hydration and drying process at 37 °C for 2 days.

The evaluation of the water content of a hydrogel is required for many biomedical applications. The percentages of water in the hydrogels were similar to each other, independently from the gelatin content (Figure 2). Moreover, the moisture values for both hydrogels were above 90%, as reported in the literature for other hydrogels [13,32].

#### 2.2.3. Swelling Test

**Figure 3:**
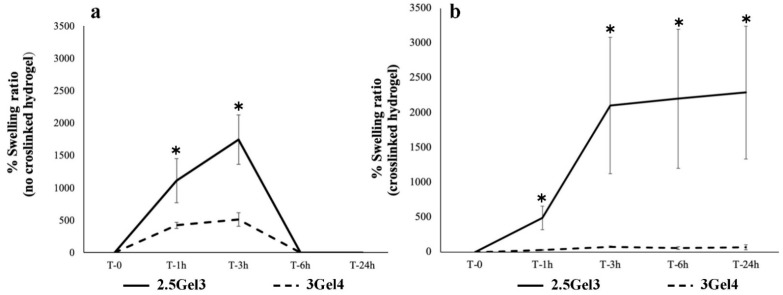
Swelling ratio of rehydrated 2.5Gel3 and 3Gel4 hydrogels by immersing them in deionized water and weighed at different time points, from 0 to 24 h. (**a**) Swelling rate of non-crosslinked hydrogels and (**b**) swelling rate of crosslinked; * *p* < 0.01.

**Figure 4:**
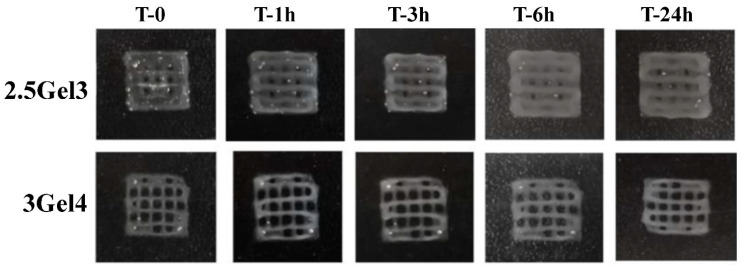
Digital photos of the 2.5Gel3 and 3Gel4 hydrogels crosslinked with 0.3 v/v% glutaraldehyde during the swelling test at different time points, from 0 to 24 h.

The swelling tests were carried out on 2.5Gel3 and 3Gel4 printed hydrogels crosslinked and non-crosslinked. These prints containing 2.5 w/v% and 3 w/v% of gelatin, after one hour of submersion in deionized water, showed a swelling rate of 1110.25% ± 339 and 423.17% ± 47.34 respectively (*p*-value 0.02543), increasing after three hours of submersion until the sixth hour, where all the prints resulted in being completely dissolved (Figure 3a). On the other hand, crosslinked prints using 0.3% glutaraldehyde immersion for 3 h showed more stable and more resistant swelling in deionized water (Figure 3b). The swelling rate of the hydrogel with 2.5 w/v% of Gel was of 489.70 ± 168.57% compared to 30.27 ± 7.59% (*p*-value 0.009199) of 3 w/v% Gel content. In Figure 3b, it is possible to observe that, after 24 h, the 2.5Gel3 had achieved a much higher swelling rate than hydrogel 3Gel4. Furthermore, the different crosslinked hydrogels were observed under the light microscope (Figure 4). The images reported in Figure 4 confirm that the crosslinked prints containing 3 w/v% Gel are more resistant to swelling.

#### 2.2.4. Hydrolysis

**Figure 5:**
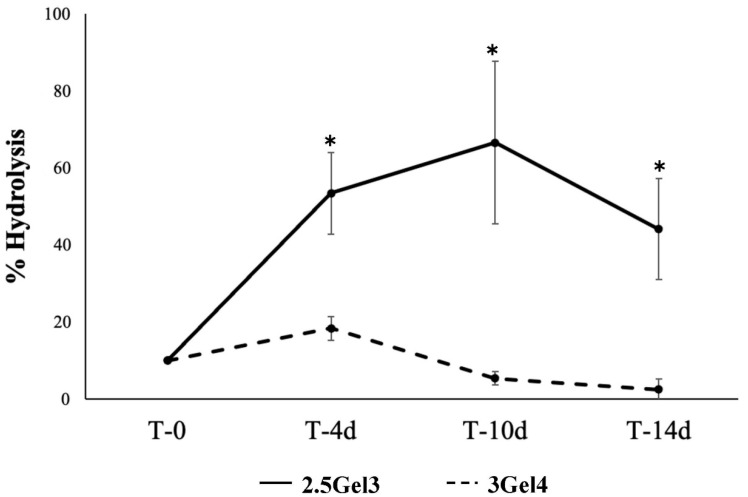
Hydrolysis percentage of the 2.5Gel3 and 3Gel4 after soaking in deionized water at 37 °C from 4 to 14 days; * *p* < 0.01.

Hydrolysis values were obtained after 14 days of the printed hydrogels’ submersion, which is probably not enough time to demonstrate the full power of water lysis [24]. Although composed mainly of water, hydrogels may undergo degradation by water, as warm temperatures and prolonged periods of time can allow water molecules to interact with the hydrogel. The hydrolysis test was not performed on non-crosslinked prints, as the high solubility of gelatin in warm water causes rapid disintegration. However, the analysis of the crosslinked prints showed initial degradation for the 3Gel4 hydrogel after 10 days with a percentage of hydrolysis of 53.96 ± 18.01% (*p*-value 0.007441) and after 14 days of 24.41 ± 28.16% (*p* = 0.005756) in water at 37 °C (Figure 5), while crosslinked 2.5 w/v% gelatin hydrogel did not lose mass in warm water.

#### 2.2.5. Porosity

**Figure 6:**
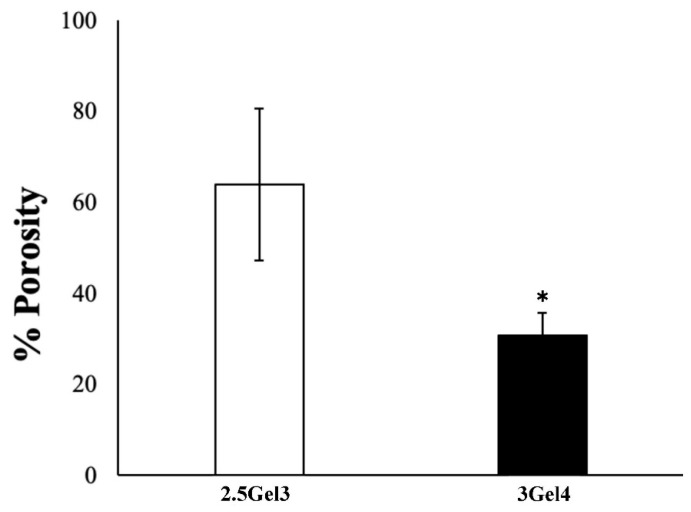
Porosity analysis of the 2.5Gel3 and 3Gel4 hydrogels carried out by liquid displacement method; * *p* < 0.01.

**Figure 7:**
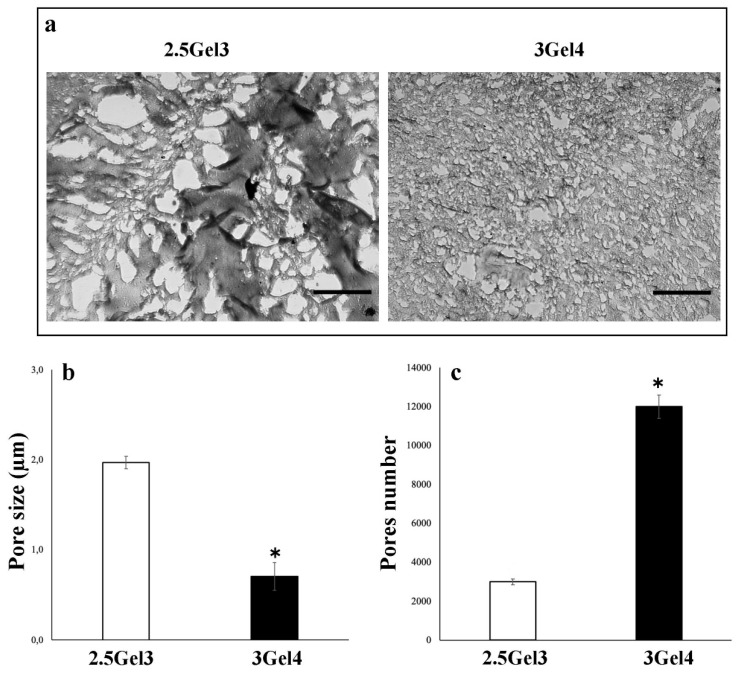
(**a**) Optical microscope representative images of the 2.5Gel3 and 3Gel4 hydrogels for the pores’ morphological analysis. Image area = 25 µm^2^, scale bar 1 µm; (**b**) average pore size (µm) of the 2.5Gel3 and 3Gel4 hydrogels and (**c**) average pores number, as analyzed by ImageJ software; * *p* < 0.01.

An initial porosity analysis, using the liquid displacement method (Figure 6), showed that the hydrogel containing 3 w/v% gelatin had a porosity of 30.82 ± 4.93%, while the 2.5 w/v% gelatin hydrogel showed a porosity value of 64.04 ± 16.68% (*p* = 0.02974). These results were compared with those obtained by morphological analysis of both hydrogels (Figure 7). Microscope images showed that the pore size in the 2.5Gel3 was greater than that observed in 3Gel4 (Figure 7b). Therefore, the morphological analysis does not confirm the results obtained by the liquid displacement method (Figure 6), thus suggesting that the 2.5 w/v% Gel appeared more porous, as the amount of ethanol embedded was high because of the larger pore size than in 3Gel4 (Figure 7b). Moreover, the number of pores within a hydrogel is an important feature for cell encapsulation (Figure 7c). Finally, the morphological analysis showed that the 3Gel4-printed hydrogel had a larger number of pores with a smaller size compared to that of 2.5Gel3, indicating that it could be used for cell encapsulation.

#### 2.2.6. Enzymatic Degradation

**Figure 8:**
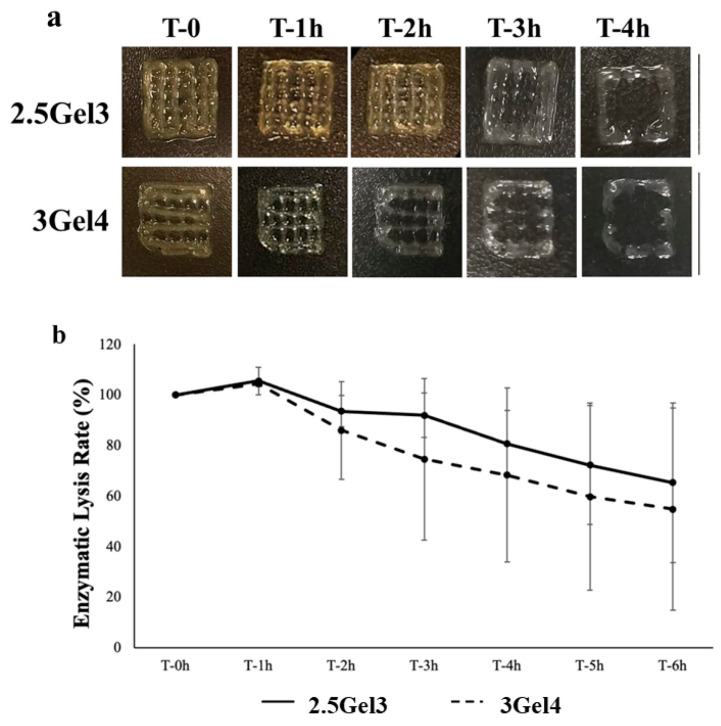
(**a**) Digital photos of enzymatic degradation analysis of the 2.5Gel3 and 3Gel4 hydrogels with collagenase I from 0 to 4 h; (**b**) enzymatic lysis rate of the 2.5Gel3 and 3Gel4 hydrogels at different time points, from 0 to 6 h.

Collagenase I is a common mammalian enzyme which is often used at a concentration of 0.1% in cell analyses, including protein digestion and tissues separation [24]. Collagenase I was used for the enzymatic degradation test, and the data obtained suggested that degradation occurs after an initial period of partial swelling (after 1 h), followed by slow and steady degradation (Figure 8a). After 2 h, the residual mass of the 2.5Gel3-printed hydrogel was 93.47 ± 6.21%, while the 3Gel4 hydrogel’s residual mass was 85.90 ± 19.43%. The degradation continued slowly over time, with no significant differences between the degradation rate of the 3Gel4 and 2.5Gel3 hydrogel (Figure 8b).

#### 2.2.7. Cell Culture

**Figure 9:**
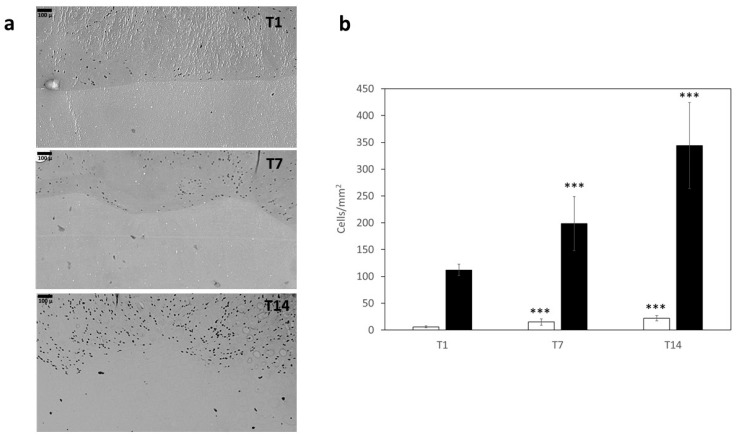
(**a**) Images obtained by Pannoramic Midi microscope slide digitizer of skin-like model with cell laden 3Gel4 (upper part) and 2.5Gel3 (lower part) after 1 (T1), 7 (T7) and 14 (T14) incubation days. Scale bar = 100 µm. (**b**) Bar-graph showing cell numbers scored in 2.5Gel3 (white bar) and 3Gel4 (black bar) at different time points; *** *p* < 0.001 compared to T1.

Figure 9a shows images of a skin-like structure that was obtained by printing 3Gel4 loaded with human keratinocytes to mimic epidermal layer (upper part) and 2.5Gel3 loaded with human fibroblast to mimic dermal layer (lower part). As shown in the images obtained on day 1 after printing (T1), the cells displayed no signs of cell death, and, as indicated in Figure 9, they increased their number after 7 (T7) and 14 (T14) days both in 3Gel4 and 2.5Gel3.

## 3. Discussion

Three-dimensional bioprinting has drown a lot of attention in the field of modern medicine for tissue engineering and wound healing. Currently, most in vitro studies have been performed on 2D or 3D cell-culture models that are devoid of the structural complexity and function of the native tissues that can be produce by using bioprinting. In fact, bioprinting offers a potential route to generate complex 3D biological constructs that are capable of mimicking the microenvironment of the native tissue and promoting physiologically relevant cell–cell and cell–matrix interactions.

Shawan et al. [13] described the synthesis of Gel/Xnt hydrogels by using 3 w/v% or 5 w/v% of gelatin and 0.6 w/v% xanthan gum and glutaraldehyde (0.5 v/v%) used as crosslinker, and they tested Gel/Xnt hybrid composite hydrogels in experimental skin burn wounds in rats as effective wound-dressing materials. However, the bioprintability properties of the obtained hybrid composite hydrogels were not evaluated. In fact, the original hydrogel could not be 3D-printed, possibly due to the low concentration of the Xnt resulting in low viscosity.

Therefore, to increase the viscosity of the new Gel/Xnt hydrogels (Table 1), the evaporation of the solution was gradually induced, instead of adding water during the synthesis, as reported by Shawan et al. [13], as well as lowering the processing temperature (from 85 °C to 60–70 °C). Furthermore, the print stability was achieved by adding glutaraldehyde after printing.

Gelatin is widely used in wound-healing applications [33,34,35,36], but one of the limitations is its liquification at the normal temperature (37 °C) for the human body [31]. To overcome this drawback, it is possible to use stabilizers, thickeners and crosslinkers, as well as xanthan gum. This is a stabilizer and a homogenizing agent [37], which, being a powder, also influences the viscosity of the hydrogel, extending the surface area and contact points for the adhesion of water molecules within the hydrated matrix. Thus, when lower amounts of xanthan gum or gelatin were used in hydrogels (Table 1), a decrease in print-shape retention was observed. On the contrary, higher amounts of gelatin and xanthan gum provided a higher shape retention, but, in addition to xanthan gum, glutaraldehyde is also needed for the gelatin crosslinking process and to maintain the print shape (Table 2). Morphologic analysis demonstrated that the amount of the xanthan gum as glutaraldehyde is important for the stabilization of the hybrid composite hydrogel. Lii et al. [38] reported that Xnt and Gel interact by hydrogen-bonding, involving the -OH groups and -NH_2_ groups of xanthan gum and gelatin, respectively, forming many bridges to which water molecules can adhere within the matrix, thus increasing the contact surface for water. In this regard, probably the concentration of 1.2 w/v% Xnt is higher than that of a low amount of gelatin (2.5 w/v%). A greater number of bonds between hydroxyl groups of the excess xanthan gum, not linked to gelatin, and water molecules cause an increase of hydration during the synthesis of the hydrogel, and therefore a lower viscosity.

Furthermore, observing the images obtained under the microscope and shown in Figure 1, we see that the non-crosslinked and crosslinked prints with glutaraldehyde respectively showed a color change from white to pale yellow, as is typically associated with the crosslinking reaction [39,40].

The human body is known to be largely composed of water. The main goal of this work is the 3D printing of the cell-seeded hydrogel during therapies such as skin grafts [41] to repair one or more layer of tissue [42] or to accelerate the wound healing. In particular, hydrogel-like materials used for wound healing should have a high percentage of water, because it could improve the interaction between cells and diffusion of molecules [13] without dehydration of the tissues. In both hydrogels, 2.5Gel3 and 3Gel4, the percentage of water is around 90%, suggesting that these materials could be used in biomedical applications.

Furthermore, swelling is also an important property to evaluate. Swelling is the ability of a hydrogel to absorb water, gaining weight and volume [25]. Hydrogels with higher swelling values are more likely to change shape and break in hydrated environments. The results of the hydrogels swelling rate may depend on the concentration of gelatin and the presence of glutaraldehyde in their composition. The crosslinking of gelatin is due to the reaction of the aldehyde functional groups present in the glutaraldehyde molecule with free non-protonated ε-amino groups (-NH_2_) of lysine or hydroxylysine belonging to the gelatin. This leads to the formation of amide linkages [43,44]. The linkages between the crosslinker and the hydrogel matrix during the crosslinking reactions affect the water-retention ability of the hydrogel [25]. This process can be observed with 3 w/v% Gel. Probably, since the functional groups of the gelatin are involved in the bonds with all aldehyde groups of the crosslinker, the latter cannot be involved in the bonds with the water molecules, thus positively influencing the swelling rate. On the other hand, when the 2.5 w/v% of Gel and 0.3 v/v% of GTA were used, the number of functional groups of Gel was not enough to bind all the aldehyde groups of the crosslinker, thus allowing a higher swelling. Comparing the swelling and hydrolysis results, we see that the crosslinked 3 w/v% gelatin hydrogel was resistant to swelling but sensible to the hydrolysis process. This could be due to a strong crosslinking of the 3Gel4 hydrogel with 0.3 v/v% GTA. For this reason, many aldehyde groups are linked to gelatin through an amide, bond and, therefore, they are less stable and more subject to the hydrolysis reaction compared to 2.5Gel3, thus allowing for a greater hydrogel degradation at high temperature by solvation and rapid disintegration of the gelatin [39,45].

The size and number of pores, their connectivity and geometry are the main characteristics for the use of the hydrogels in biomedical applications. Indeed, the ability of cells to proliferate into a 3D structure is affected by the size of the pores, as well as is the biodegradability of the structure itself [46]. In particular, to allow for vascularization and tissue formation, pore sizes should be less than 500 μm, as larger pore sizes could decrease cell–cell interaction and, thus, their proliferation [46,47]. The 3Gel4 has a greater number of smaller pores, probably due to the strong bond with the crosslinker that makes this material denser and, therefore, gives it a better porosity compared to 2.5Gel3.

The results of the enzymatic degradation and hydrolysis test showed the biodegradability of the produced hydrogel. Although crosslinked gelatin hydrogels are soft materials, they are capable of causing irritation and reactions [24]. For this reason, the synthesized Gel/Xnt hydrogel does not need to be highly resistant to degradation, thus allowing host cells to gradually replace the hydrogel in wound healing and absorbing it completely.

Finally, human fibroblast and keratinocytes were mixed with 2.5Gel3 and 3Gel4 hydrogels respectively, and a skin-like structure was printed to evaluate the biocompatibility of both materials. The images of the cell distribution and proliferation in the hydrogels collected after 1, 7 and 14 days in culture showed a good proliferation. These results are also due to the presence of numerous and small pores that induce greater cell proliferation in a 3D structure [46] and to the good biodegradability of the material composing the hydrogels.

## 4. Materials and Methods

### 4.1. Hydrogel Preparation

Shawan et al. [13] reported the preparation of the hydrogels by using different formulations of gelatin (Gel), namely 3 w/v% and 5 w/v%, and 0.6 w/v% of xanthan gum (Xnt) for heal ulcers. Based on this work, new compositions of Gel/Xnt hybrid composite hydrogels were designed, modifying the ratio between two components to obtain the best printable hydrogel. Bovine gelatin (Gel) (Sigma-Aldrich, St. Louis, MO, USA) was slowly added to deionized water at 70 °C, under magnetic stirring to avoid the formation of clumps. Afterward, xanthan gum (Xnt) (Sigma-Aldrich, St. Louis, MO, USA) was added into the gelatin solution, and the temperature was maintained between 60 and 70 °C until water evaporation. Hydrogel was then stored at 4 °C until use. The formulations used to produce the different hydrogels are reported in Table 1.

### 4.2. Bioprinting

#### Hydrogel Bioprinting

CellInk Inkredible 3D printer (CellInk Inkredible 3-D printer, CellInk, Gothenburg, Sweden) was used for the hydrogel printing. Hydrogels were printed to obtain 1 cm^2^ squares. After preparation, hydrogels were left at 4 °C from 3 to 10 days, and they were warmed to room temperature before printing. Bio-ink syringes (3 mL) were filled with the different hydrogels and then loaded into the 3D printer. Printability trials were run at different pressures (from 10 to 20 kPa), as reported in Table 1.

### 4.3. Hydrogel Crosslinking

Crosslinking trials were performed by using different concentrations of glutaraldehyde (GTA, 0.3, 0.5 and 1 v/v%,) obtained from a 25 v/v% stock solution. In addition, for each concentration, different submersion times in the glutaraldehyde solution (1 or 3 h) were tested. The crosslinking of a hydrogel structure occurs in the post-printing process. Crosslinked hydrogels were added to cell culture Dulbecco’s Modified Eagle’s medium (DMEM) to test their stability (Table 2).

### 4.4. Characterization of 3D-Printed Hydrogel

#### 4.4.1. Morphology

The macroscopic morphology of the hydrogel was observed by taking several photos, using a high-resolution digital camera (NIKON D5600). Optimal cutting temperature compound (OCT compound) was used to incorporate the hydrogels prior to frozen sectioning on a microtome cryostat, obtaining hydrogel micron slices. The optical reverse microscope (Motic AE2000, Motic Europe, Barcelona, Spain) was used to evaluate the microscopic morphology of the different printed hydrogels, both crosslinked and non-crosslinked.

#### 4.4.2. Moisture

The moisture percentage was measured as described by Shawan et al. [13]. On day 3 of stabilization, hydrated prints were weighted (Wᴴ), stored at 37 °C for 2 days to allow the drying process to be completed and then weighed again (Wᴰ). The percentage of moisture (water) in a printed hydrogel was calculated as follows (Equation (1)):

Moisture (%) = [(Wᴴ − Wᴰ)/Wᴴ] × 100(1)

where Wᴴ is original weight of the sample before drying, and Wᴰ is weight of the sample after drying. All the experiments were replicated three times, with at least three samples for each condition.

#### 4.4.3. Swelling Test

Printed hydrogel, both non-crosslinked and crosslinked, were dried at 37 °C for 48 h to perform the swelling test [25]. Dried printed hydrogels were weighed, rehydrated by soaking in deionized water and weighed again after the water was removed, at different time points (time 0, 1, 3, 6 and 24 h). The residual water was eliminated, using filter paper, by capillary action. The swelling ratio (S) [25] was calculated by using the following equation (Equation (2)):

S = [(Wᴴ − Wᴰ)/Wᴰ] × 100.(2)

where Wᴴ is sample hydrated weight, and Wᴰ is initial dehydrated weight.

All the experiments were replicated three times, with at least three samples for each condition.

#### 4.4.4. Hydrolysis

In order to evaluate the hydrolysis, crosslinked printed hydrogels were weighed (time 0) and immersed in deionized water at 37 °C. After the time point from 4 to 14 days, the weight of the crosslinked prints was taken by removing the deionized water and lightly dabbing the crosslinked prints with filter paper. The percentage of hydrolysis was obtained by using the following equation (Equation (3)):

Hydrolysis (%) = [(W^I^ − W^F^)/W^I^] × 100 (3)

where W^I^ is the before soaking weight, and W^F^ is the remaining weight after soaking and removing of the deionized water. All the experiments were replicated three times, with at least three samples for each condition.

#### 4.4.5. Porosity

The porosity of the printed hydrogels was evaluated by using the liquid displacement method [24]. Absolute ethanol, which causes neither swelling nor shrinking of gelatin [24], was used for prints’ submersion. After 5 min of submersion in a known quantity of absolute ethanol, samples were weighed. The porosity of the hydrogel was calculated as follows (Equation (4)):

Porosity (%) = [(W_1_ − W₃)/(W₂ − W₃)] × 100 (4)

where W_1_ is the initial weight of pure ethanol, W₂ is the total weight that combines the weight of the hydrogel with the weight of the ethanol and W₃ is the ending weight of ethanol without hydrogel [24].

Furthermore, Greyscale 8-bit TIFF images of the 2.5Gel3 and 3Gel4 were processed by using ImageJ software. The ImageJ “particle analysis” function was then used to calculate the size and the numerosity of hydrogel pores. All the experiments were replicated three times, with at least three samples for each condition.

#### 4.4.6. Enzymatic Degradation

The biological stability of the hydrogels was evaluated by exposing them to 0.1% collagenase type IA (>125 CDU/mg, Sigma-Aldrich, St. Louis, MO, USA) to assess degradation rates [24]. Printed hydrogels were dried at 37 °C for two days, and before the analysis, the dry prints were soaked in PBS for 1 h, and then they were exposed to the enzymatic solution (prepared in PBS 1X) at 0.1% collagenase type I for 6 h. Enzymatic degradation tests [24] were performed at 37 °C in a horizontal shaker. After different time points (1, 2, 3, 4, 5 and 6 h), the excess water was removed from the printed hydrogels and then weighed. The biomaterial degradation was determined by calculating the percentage of remaining weight versus the original weight. All the experiments were replicated three times, with at least three samples for each condition.

### 4.5. Cell Culture

Primary human-derived-skin fibroblasts and human keratnocytes spontaneously immortalized (HaCaTs) were used for testing printable hydrogels biocompatibility. HaCaTs were purchased from Cell Lines Service GmbH (Eppelheim, Germany), while human fibroblasts were a kind gift from Professor Marco De Andrea (University of Turin, Turin, Italy). Both cell types were grown in culture flask in Dulbecco’s modified Eagle’s medium (DMEM) containing high glucose levels supplemented with 10% heat-inactivated fetal bovine serum (FBS) and 1% Penicillin–Streptomycin (all from Immunological Science, Rome, Italy) in a humidified atmosphere containing 5% CO_2_ at 37 °C. Two different cell populations (keratinocytes and fibroblasts) inserted into the two hydrogels of different porosity were used for printing a simple 3D skin-like model with a surface layer that mimics the epidermis (high cell concentration for the epithelium) and a lower layer that mimics the dermis (low cellular concentration for the connective). The 2.5Gel3 and 3Gel4 printable hydrogels were gently mixed with 0.5 × 10^6^ fibroblasts/mL and 5 × 10^6^ HaCaT cells/mL, respectively, using two syringes connected under a sterile cap. Small cylinders (0.5 cm high, 1 cm wide) were printed by using cell-laden 2.5Gel3 for the lower layers and 3Gel4 for the upper layers. The cylinders were crosslinked by using GTA as previously indicated. The cell-laden cylinders were washed three times with sterile PBS and incubated for 1, 7 and 14 days in DMEM 10% FBS at 37 °C in a 5% CO_2_ atmosphere. At the end of the incubation time, the culture medium was removed, and the hydrogels containing the cells were washed with phosphate buffered saline (PBS) 3 times, formalin fixed and paraffin embedded. Samples were then cut in 5μ slices and stained with hematoxylin/eosin; the cells grown in hydrogels were observed by using a digitizer Pannoramic Midi for microscope slides (Epredia, Palermo, Italy), and their number was evaluated by using the ImageJ software. All the experiments were replicated three times, with at least three samples for each condition.

### 4.6. Statistical Analysis

Data were presented as mean ± standard deviation (SD). Statistical analyses were performed by using GraphPad PRISM software. Kolmogorov–Smirnoff test was applied to verify the normal distribution of data. One-way ANOVA was employed for multiple comparison, the whereas t-test was used when a comparison between two group occurred. The *p*-values < 0.05 were considered statistically significant.

## 5. Conclusions

The hybrid composite hydrogels containing different percentages of gelatin and the same amount of xanthan gum were designed to obtain printable materials for in vitro and in vivo biomedical applications. Among all the hydrogel formulations modulating the process and the parameters of the 3D printer, the 2.5 w/v% and 3 w/v% of gelatin with 1.2 w/v% xanthan gum resulted in being printable, with a good resolution. The characterization results showed that 3Gel/4hydrogel crosslinked with 0.3 v/v% GTA presents optimal properties compared to the hydrogel with 2.5Gel3. In particular, the 3Gel4 hybrid composite hydrogel was able to maintain its print shape, to resist to the swelling and to degrade easily. Moreover, it shares a very good biocompatibility with the 2.5Gel3, and it could be used as a matrix for cell growth, for the development of in vitro tissues or also for wound dressing, allowing the host cells to gradually replace the hydrogel with a secreted matrix.

## Figures and Tables

**Table 1:** Hydrogels compositions.

Label	Composition (w/v%)		Printability (Qualitative Evaluation)
	Gelatin (Gel)	Xanthan Gum (Xnt)	
2.5Gel1	2.5	0.7	−
2.5Gel2	”	1	+
2.5Gel3	”	1.2	++
3Gel1	3	0.3	−
3Gel2	”	0.7	−
3Gel3	”	1	+
3Gel4	”	1.2	++

**Table 2:** Different concentrations of glutaraldehyde and crosslinking times for 2.3Gel3 and 3Gel4.

Label	Composition (w/v%)		GTA Concentration (v/v%)	Time (h)	Stability in DMEM
	Gelatin (Gel)	Xanthan Gum (Xnt)			
2.5Gel3	2.5	1.2	0.3	1	−
”	”	”	”	3	++
”	”	”	0.5	1	−
”	”	”	”	3	−
”	”	”	1	1	−
”	”	”	”	3	−
3Gel4	3	1.2	0.3	1	−
”	”	”	”	3	++
”	”	”	0.5	1	−
”	”	”	”	3	−
”	”	”	1	1	−
”	”	”	”	3	−

## Data Availability

The raw/processed data required to reproduce these findings cannot be shared at this time, as the data also form part of an ongoing study.
