# Correction: Piola et al. 3D Bioprinting of Gelatin–Xanthan Gum Composite Hydrogels for Growth of Human Skin Cells. *Int. J. Mol. Sci.* 2022, *23*, 539

**DOI:** 10.3390/ijms25179402

**Published:** 2024-08-29

**Authors:** Beatrice Piola, Maurizio Sabbatini, Sarah Gino, Marco Invernizzi, Filippo Renò

**Affiliations:** 1 Innovative Research Laboratory for Wound Healing, Health Sciences Department, Medical School, Università del Piemonte Orientale, Via Solaroli 17, 28100 Novara, Italy; 20021634@studenti.uniupo.it (B.P.); sarah.gino@uniupo.it (S.G.); 2 Department of Sciences and Technological Innovation, Università del Piemonte Orientale, Via T. Michel 11, 15121 Alessandria, Italy; maurizio.sabbatini@uniupo.it; 3 Health Science Department, Physical Medicine and Rehabilitation Division, Università del Piemonte Orientale, Via Solaroli 17, 28100 Novara, Italy; marco.invernizzi@med.uniupo.it; 4 Department of Integrated Research and Innovation, Translational Medicine Unit (DAIRI), Hospital “S.S. Antonio e Biagio e Cesare Arrigo”, 15121 Alessandria, Italy

In the original publication [1], there was a mistake published in Figure 8a. In particular, for the images relating to the 2.5Gel3 hydrogel at times 1 h and 2 h, images of samples of the 3Gel4 hydrogel were mistakenly used. The corrected images for hydrogels 2.5Gel3 have been now added. The corrected Figure 8a appears below. The authors state that the scientific conclusions are unaffected. This correction was approved by the Academic Editor. The original publication has also been updated.

## Figures and Tables

**Figure 8:**
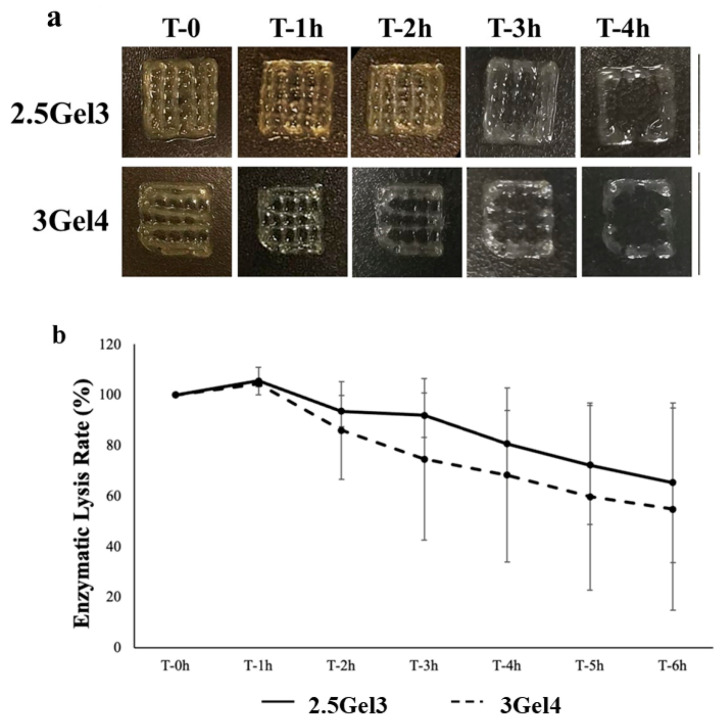
(**a**) Digital photos of enzymatic degradation analysis of the 2.5Gel3 and 3Gel4 hydrogels with collagenase I from 0 to 4 h; (**b**) enzymatic lysis rate of the 2.5Gel3 and 3Gel4 hydrogels at different time points, from 0 to 6 h.
